# Surface roughness and oxygen inhibited layer control in bulk-fill and conventional nanohybrid resin composites with and without polishing: in vitro study

**DOI:** 10.1186/s12903-022-02297-w

**Published:** 2022-06-26

**Authors:** Andrea Gaviria-Martinez, Leonor Castro-Ramirez, Marysela Ladera-Castañeda, Luis Cervantes-Ganoza, Hernán Cachay-Criado, María Alvino-Vales, Goretty Garcia-Luna, Carlos López-Gurreonero, Alberto Cornejo-Pinto, César F. Cayo-Rojas

**Affiliations:** 1 School of Stomatology, Universidad Privada San Juan Bautista, Lima, Peru; 2 “Grupo de Investigación Salud y Bienestar Global”, Postgraduate School, Universidad Nacional Federico Villarreal, Lima, Peru; 3 Faculty of Stomatology, Universidad Inca Garcilaso de la Vega, Lima, Peru

**Keywords:** Bulk-fill resin, Comparative study, Dental materials, Dental polishing, Dentistry, Nanohybrid resin, Oxygen inhibited layer, Resin composite, Surface roughness

## Abstract

**Background:**

It has been demonstrated that dental restorations with rough surfaces can have several disadvantages such as pigment retention or plaque accumulation, which can facilitate caries formation, color variation, loss of brightness, degradation of restoration, among others. The present study aimed to assess surface roughness in bulk fill and conventional nanohybrid resins with and without polishing, controlling the oxygen inhibited layer.

**Methods:**

This in vitro and longitudinal experimental study consisted of 120 resin blocks of 6 mm diameter and 4 mm depth, divided into two groups: Bulk Fill (Tetric^®^ N-Ceram Bulk-fill, Opus Bulk Fill APS, Filtek™ Bulk Fill) and conventional nanohybrid (Tetric^®^ N-Ceram, Opallis EA2, Filtek™ Z250 XT). Each resin group was divided into two equal parts, placing glycerin only on one of them, in order to control the oxygen inhibited layer. Subsequently, the surface roughness was measured before and after the polishing procedure with Sof-Lex discs. The data were analyzed with the T-test for related measures, and for comparison between groups before and after polishing, the non-parametric Kruskal Wallis test with the Bonferroni post hoc was used, considering a significance level of *p* < 0.05.

**Results:**

Before polishing, the resin composites with the lowest surface roughness were Opus Bulk Fill APS (0.383 ± 0.186 µm) and Opallis EA2 (0.430 ± 0. 177 µm) with and without oxygen inhibited layer control, respectively; while after polishing, those with the lowest surface roughness were Opus Bulk Fill APS (0.213 ± 0.214 µm) and Tetric N-Ceram (0.097 ± 0.099 µm), with and without oxygen inhibited layer control, respectively. Furthermore, before and after polishing, all resins significantly decreased their surface roughness (*p* < 0.05) except Opus Bulk Fill APS resin with oxygen inhibited layer control (*p* = 0.125). However, when comparing this decrease among all groups, no significant differences were observed (*p* < 0.05).

**Conclusion:**

The Opus Bulk Fill APS resin with oxygen inhibited layer control presented lower surface roughness both before and after polishing, being these values similar at both times. However, after polishing the other bulk fill and conventional nanohybrid resins with and without oxygen inhibited layer control, the surface roughness decreased significantly in all groups, being this decrease similar in all of them.

**Supplementary Information:**

The online version contains supplementary material available at 10.1186/s12903-022-02297-w.

## Background

Resin composites continue to be the most widely used material in dental practice because technology has been improving their mechanical and optical properties in order to achieve highly esthetic and functional restorations [1–4].

Resin composites have in their structure an organic matrix with a mixture of monomers such as Bis-GMA (Bisphenol-A-Glycidyl Methacrylate), TEGDMA (Triethylene Glycol Dimethacrylate), UDMA (Urethane Dimethacrylate), HEMA (Hydroxyethylmethacrylate), Bis-EMA (Bisphenol A Polyethylene Glycol Diether Dimethacrylate), fillers such as silica, quartz or ceramic glass and a photoinitiator such as camphorquinone, BAPO (bisacyl phosphine oxide), among others, thus obtaining a classification of macrohybrid, microhybrid, nanohybrid and hybrid, which vary the quantity and size of their particles [3, 5–7]. However, the increase in filler loading also leads to an increase in stiffness and stress during light curing [3]. For this reason, a new resin composite system called “Bulk Fill or monoincremental” was developed, which can be placed in increments of 4 mm, thus reducing the number of clinical steps and the shrinkage effect, as well as having polymerization accelerators in its composition that reduce light curing time [4, 8].

Because resin composites are highly esthetic, they are the first choice for restoring teeth. Therefore, their shelf life continues to be a concern. It has been reported that one of the factors contributing to clinical success of resin composites is the final polishing of restoration, since it allows to obtain a smooth and shiny surface [4, 9]. In this sense, it has been demonstrated that a rough surface generates several complications over time, such as pigment retention and plaque accumulation, which would facilitate the formation of secondary caries, restoration degradation and gingival inflammation [4, 9, 10]. Likewise, the lack of a smooth finish in the occlusal contact areas would generate greater friction, causing wear on the antagonist tooth surface and even microfractures in the restoration. [4, 10]

On the other hand, polishing quality and surface finish in resin composites is influenced by several factors such as filler particle size and filler loading [9, 11, 12]. Some studies indicate that to achieve ideal polishing it is necessary for resin composites to have small particles, so microfilled resin composites achieve better surface quality and higher gloss [9, 12]. However, these microfilled resin composites have inferior mechanical properties compared to universal resin composites such as nanohybrids and nanofillers [12].

To test the effectiveness of different polishing systems on resin composites, it is common to assess surface roughness. Several studies report that aluminum abrasive polishing wheel produces better results for most types of resin composites compared to other polishing tools [13–15].

Although finishing and polishing systems help to avoid a rough resinous surface, it is still a challenge to completely remove the oxygen-inhibited layer (OIL), which forms during light-curing of resin composite. Upon contact with atmospheric oxygen, the resin composite leaves an uncured layer because oxygen inhibits the polymerization reaction, resulting in formation of a polymer chain that is more prone to staining and wear [2, 10]. In order to achieve a highly esthetic and functional restoration, it is necessary to block OIL at the time of light curing, since it decreases the surface quality of restoration [2, 16]. Many dentists use glycerin to prevent the formation of OIL, since it prevents atmospheric oxygen from contacting the resin composite surface, thus preventing it from reacting with free radicals, improving the degree of conversion and the surface mechanical properties of resin composites [10, 16, 17].

Different studies had as limitations the operator variable, the types of movement and the pressure applied for polishing, since these can influence surface roughness, as reported by St-Pierre et al. [12] Babina et al. [18] and Madhyastha et al*.* [19]. Due to this, all suggested that procedures should be performed by one operator to reduce biases, so the need arises to assess surface roughness using polishing systems with identical movements, in the same direction and performed by a single operator. In addition, studies such as Aljamhan et al. [20] and Khudhur et al. [21] recommended measuring surface roughness before polishing, since they only measured and compared surface roughness between different resin composites after polishing, and were unable to assess the variation between before and after polishing. In turn, Ramírez et al. [10] and Ishii et al. [4] suggested assessing the surface characteristics of bulk fill resin composites versus conventional nanohybrid resin composites.

Therefore, the present study aimed to assess surface roughness of bulk fill and conventional nanohybrid resin composites with and without polishing, controlling the oxygen inhibited layer. Specific objectives were: (1) To determine surface roughness, before and after polishing, of bulk fill and conventional nanohybrid type resin composites, with and without oxygen inhibited layer control. (2) To compare surface roughness, before and after polishing, of bulk fill and conventional nanohybrid type resin composites, with and without oxygen inhibited layer control. (3) To compare surface roughness variation between before and after polishing of bulk fill type and conventional nanohybrid type resin composites, with and without oxygen inhibited layer control.

The null hypothesis stated that there was no significant difference in surface roughness of bulk-filled resin composites versus conventional nanohybrid resin composites, with and without polishing, after control of the oxygen inhibited layer. This study considered the CRIS Guidelines (Checklist for Reporting In-vitro Studies) [22].

## Methods

### Type of study and delimitation

This longitudinal and prospective in vitro experimental study was conducted at the School of Stomatology of the Universidad Privada San Juan Bautista and at the High Technology Laboratory Certificate (ISO/IEC Standard: 17,025), Lima, Peru, in the months of October to December 2021, with approval letter No.1199-2021-CIEI-UPSJB.

### Sample calculation and selection

**Fig. 1:**
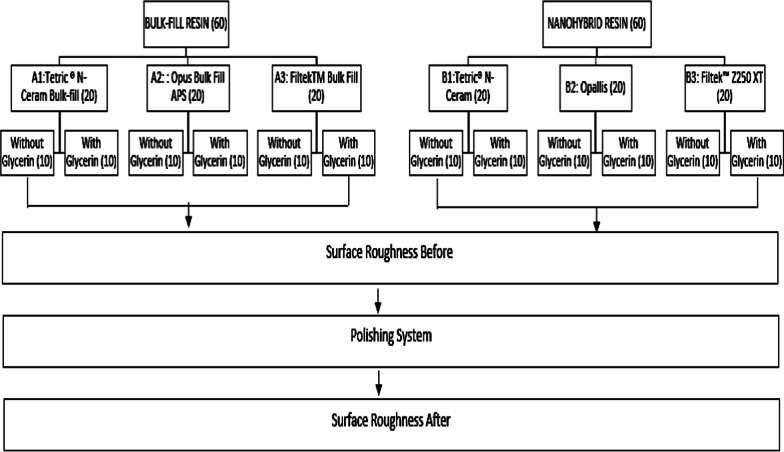
Random distribution of groups according to type of resin composite, use of glycerin and type of polishing

A total of 120 resin composite blocks were made and standardized, evenly distributed in six groups of 20 blocks. They were then divided in simple random order without replacement into two equal subgroups of resin composite blocks with glycerin (*n* = 10) and without glycerin (*n* = 10) (Fig. 1). The total sample size (*n* = 120) was calculated from data obtained in a previous pilot study in which the variance analysis formula was applied in the statistical software G*Power version 3.1.9.7 considering a significance level (α) = 0.05 and a statistical power (1-β) = 0.80, with an effect size of 0.13, with 12 groups and 2 paired measures.

### Variables

Variables included were: type of compact resin composite, surface roughness, polishing system and glycerin application.

### Sample characteristics and preparation

**Fig. 2:**
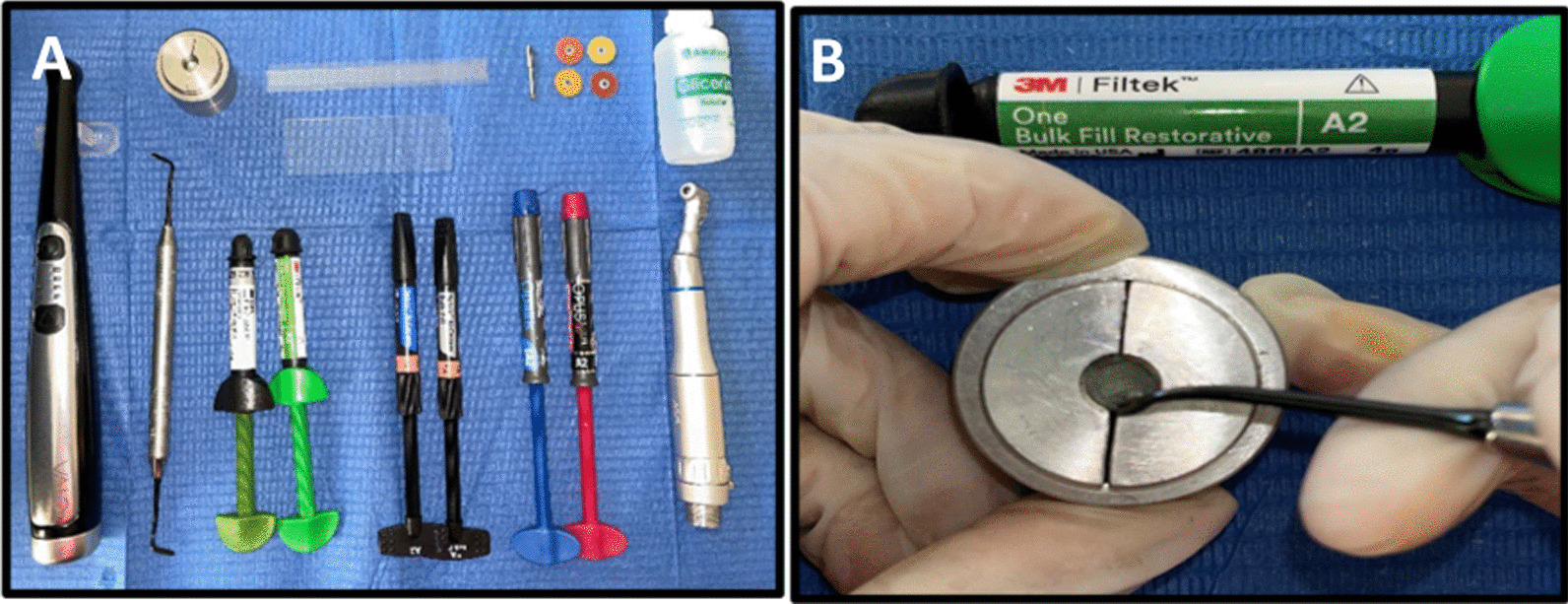
**A** Materials and instruments used. **B** Compaction of resin composite inside the stainless-steel mold

The samples were 120 blocks of bulk fill and conventional nanohybrid resin composites measuring 6 mm in diameter and 4 mm in depth [10, 23]. (Table 1). The resin composite blocks were made by a single operator, coded and distributed in the following way (Fig. 2):

**Table 1:** Materials tested

Product	Type	Composition	Filler % (wt-vol)	Manufacturer	Lot
Filtek™ bulk fill A2	Nanofill bulk fill	Matrix: AUDMA, UDMA, AFM y 1, 12-dodecane-DMA Filler: not agglomerated/not aggregated silica, not agglomerated/not aggregated zirconia, aggregated zirconia / silica compound, ytterbium trifluoride	76.5 wt-58.4 vol	3 M, ESPE, St. Paul, MN, USA	NC74349
Tetric^®^ N-ceram bulk-fill IVA	Nanohybrid bulk fill	Matrix: bis-GMA, bis-EMA, UDMA Filler: barium silicate alumino glass, “isofiller” (prepolymer, glass and ytterbium fluoride), ytterbium fluoride and mixed oxides	76 wt-54 vol	Ivoclar Vivadent, Schaan, Liechtenstein	Z02TBZ
Opus bulk fill APS A2	Nanohybrid Bulk Fill	Matrix: UDMA Filler: Nanofiller Photoinitiating-Advanced Polymetization System (APS). Inorganic load of silanized silicon dioxide (sílica), barium glass aluminosilicate	76.5 wt-58.4 vol	FGM, Santa Catarina, Brasil	010,221
Opallis EA2	Nanohybrid	Matrix: Bis-GMA, Bis-EMA, UDMA, TEGDMA. Filler: The loads are a combination of silanized barium-aluminum silicate glass and nanoparticles of silicon dioxide, camphorquinone as photoinitiator, accelerators, stabilizers and pigments	79.8 wt-58 vol	FGM, Santa Catarina, Brasil	171,120
Tetric^®^ N-ceram A2	Nanohybrid	Matrix: Bis-GMA, Bis-EMA, UDMA Filler: Dimethacrylates, additives, catalysts, stabilizer sand pigments, barium glass, ytterbium trifluoride, mixed oxide and prepolymerized filler	81 wt-57 vol	Ivoclar Vivadent, Schaan, Liechtenstein	Z022ZP
Filtek™ Z250 XT A2	Nanohybrid	Matrix: BIS-GMA, TEGDMA, UDMA Filler: Silane treated ceramic, Bisphenol a polyethylene glycol diether dimethacrylate	82 wt-68 vol	3 M, ESPE, St. Paul, MN, USA	NE65758
Sof-lex System	Finishing polishing sytem	Aluminum oxide abrasive discs	–	3 M, ESPE, St. Paul, MN, USA	NA38805 NC80025 NA38805 NC93054

**Fig. 3:**
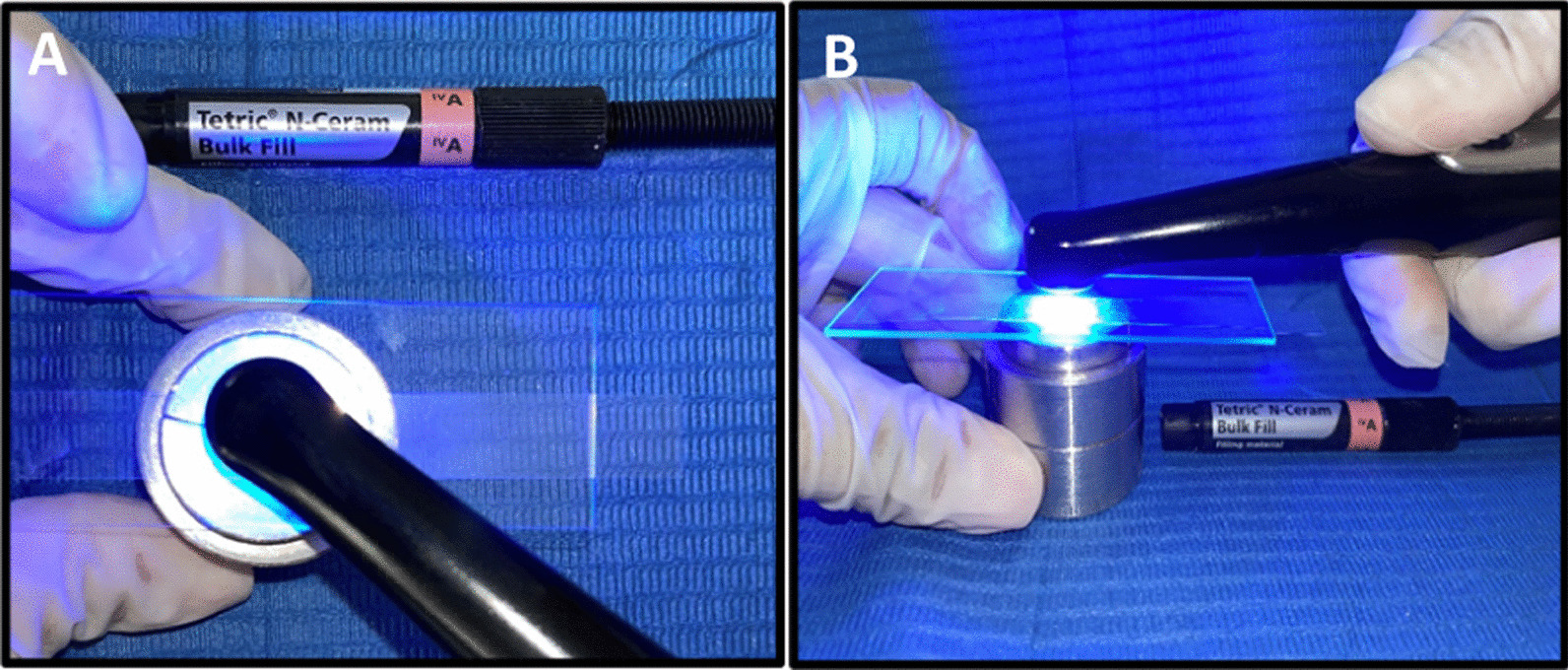
**A** Celluloid matrix and 1 mm slide. **B** Light curing of resin composite

**Fig. 4:**
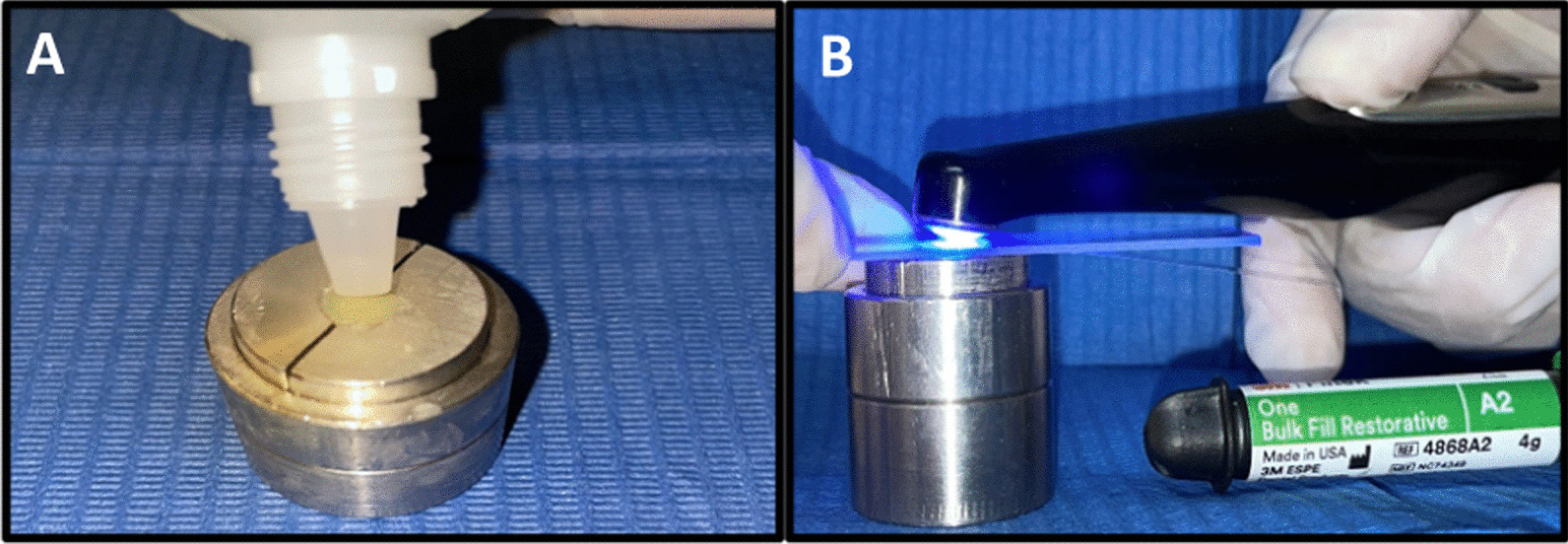
**A** Glycerin application prior to light curing. **B** Light curing of resin composite

For non-glycerin applied and unpolished groups (control groups), a celluloid matrix was placed on top of the mold and a 1 mm thick microscope slide on top of the matrix to ensure that upper and lower surfaces were parallel. The resin composite samples were light-cured from the top of the mold with an LED (Light-Emitting Diode) curing lamp (Valo^®^, Ultradent©, South Jordan, UT, USA) with an intensity of 1000 mW/cm^2^ for 20 s (Fig. 3). The intensity was verified by a radiometer (Litex 682, Dentamerica^®^, City of Industry, CA, USA). For glycerin-applied and unpolished groups, the same procedure was followed, except that before light-curing the last increment, a layer of glycerin was applied on the surface of sample and light-cured from top of the mold with the same intensity and time. (Fig. 4).

**Fig. 5:**
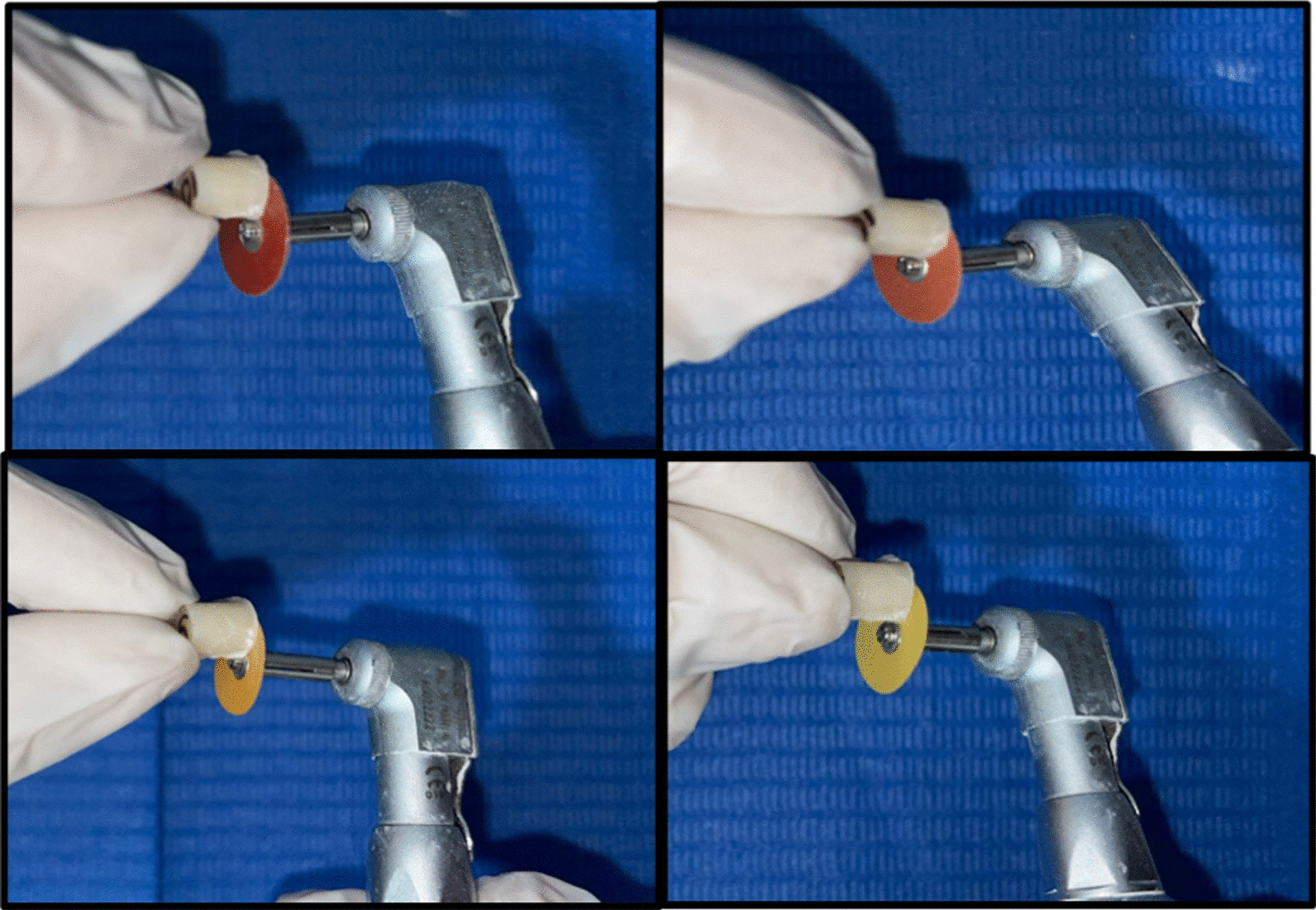
Four-step polishing procedure with Sof-lex system

For non-glycerin applied and polished groups, a celluloid matrix was placed on top of the mold and a 1 mm thick microscope slide was placed on top of the matrix to ensure that upper and lower surfaces were parallel. The resin composite layers were light-cured from top of the mold with an LED curing lamp at an intensity of 1000 mW/cm^2^ for 20 s. Subsequently, the specimen surfaces were polished by the same operator with a four-step disc system (Sof-Lex, 3 M ESPE, St. Paul, SM, USA) from coarse to fine grit (Table 1). The polishing discs were changed after use on each sample. For glycerin-applied and polished groups, the same procedure was followed except that before light-curing the last increment, a layer of glycerin was applied to the sample surface, then light-cured from top of the mold with the same intensity and time, and finally polished under the same system. (Fig. 5).

### Surface roughness test

Surface roughness was measured on 120 resin composite blocks before the polishing procedure was performed. After that, the sample was stored in an oven at 37 °C for 24 h. Then, the upper surface of the resin composite blocks, which was previously marked, was polished according to the type of treatment assigned to each group and the surface roughness was measured again. On each resin block the measurements were performed with the 0.001 µm roughness meter (Huatec SRT-6200^®^, Haidian, Beijing, China). For measuring the surface roughness values of samples, the measuring length was taken as 1.75 mm and the shear value as 0.25 mm.

**Fig. 6:**
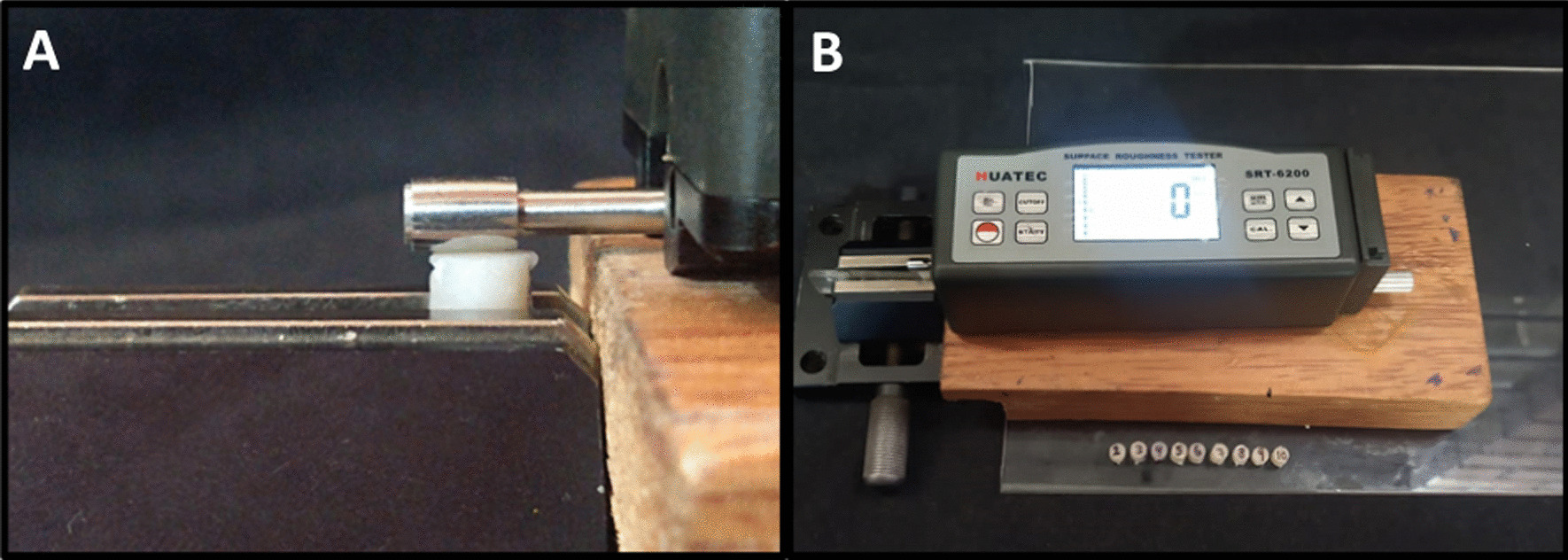
**A** Surface roughness measurement. **B** HUATEC SRT-6200 Roughness Tester

The surface roughness value on each resin composite block was determined as the average in microns of the measurements on four different areas of the upper surface. (Fig. 6).

### Statistical analysis

Data collected were recorded in a Microsoft Excel 2019^®^ spreadsheet and subsequently imported for statistical analysis by the SPSS program (Statistical Package for the Social Sciences Inc. IBM, NY, USA) version 24.0. For descriptive analysis, measures of central tendency and dispersion, such as mean and standard deviation, were used. For hypothesis testing, we evaluated if the data presented normal distribution and homoscedasticity, using Shapiro Wilk’s test and Levene’s test, respectively. According to these results, normal distribution was observed in the mean difference for all groups (before and after polishing), so it was decided to use the T-test for related measures. However, for intergroup comparison, both before and after polishing, the nonparametric Kruskal Wallis test with Bonferroni’s post hoc was used. A significance level of *p* < 0.05 was considered for all comparisons.

## Results

**Fig. 7:**
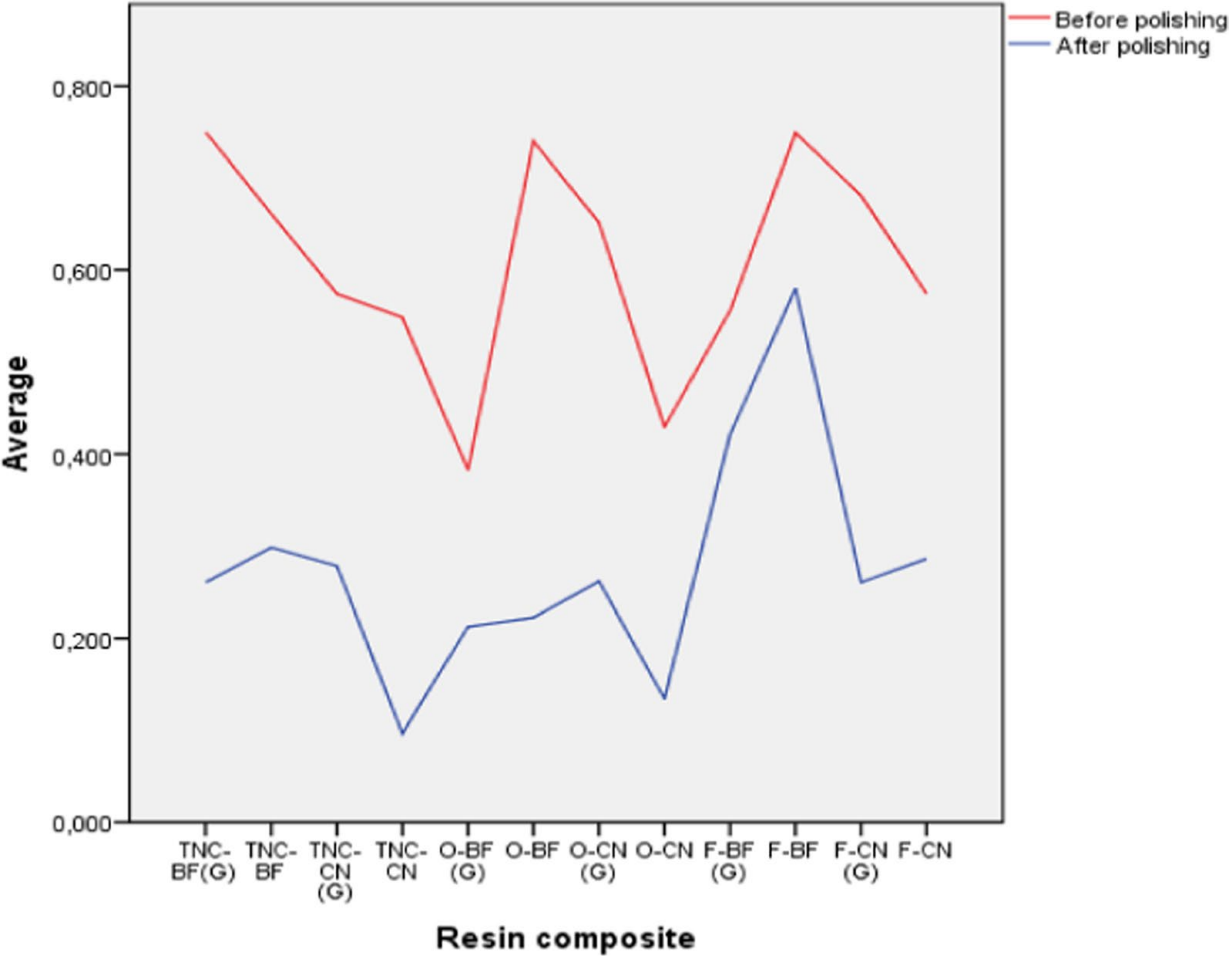
Average surface roughness before and after polishing of resin composites with and without oxygen inhibited layer control

Before polishing, it could be observed that the resin composites with highest surface roughness were Tetric N-Ceram Bulk Fill (0.750 ± 0.380 µm) and Filtek Bulk Fill (0.749 ± 0. 433 µm), with and without oxygen inhibited layer control, respectively. The resin composites with lowest surface roughness were Opus Bulk Fill APS (0.383 ± 0.186 µm) and Opallis EA2 (0.430 ± 0.177 µm), with and without oxygen inhibited layer control, respectively (Table 2). On the other hand, after polishing it could be observed that the resin composites with highest surface roughness was Filtek Bulk Fill with control (0.422 ± 0.231 µm) and without control (0.580 ± 0. 398 µm) of the oxygen inhibited layer; while the resin composites with lowest surface roughness were Opus Bulk Fill APS (0.213 ± 0.214 µm) and Tetric N-Ceram (0.097 ± 0.099 µm), with and without control of the oxygen inhibited layer (Table 2). In addition, it could be seen that all resin composites without exception decreased their surface roughness after being subjected to polishing (Fig. 7) (Additional file 1: Table S1).

**Table 2:** Descriptive values of surface roughness before and after polishing of bulk fill and conventional nanohybrid resin composites, with and without oxygen inhibition layer control

Polish	Resin composite	*n*	Mean	SD	Median	IQR	Minimum	Maximum
Before	TNC-BF(G)	10	0.750	0.380	0.547	0.692	0.403	1.441
	TNC-BF	10	0.661	0.482	0.503	0.740	0.120	1.498
	TNC-CN (G)	10	0.574	0.342	0.466	0.477	0.264	1.288
	TNC-CN	10	0.549	0.315	0.581	0.572	0.112	1.056
	O-BF (G)	10	0.383	0.186	0.374	0.332	0.113	0.629
	O-BF	10	0.740	0.431	0.669	0.830	0.085	1.340
	O-CN (G)	10	0.651	0.524	0.514	0.483	0.155	1.899
	O-CN	10	0.430	0.177	0.442	0.285	0.144	0.725
	F-BF (G)	10	0.556	0.233	0.462	0.267	0.364	1.038
	F-BF	10	0.749	0.433	0.660	0.711	0.294	1.555
	F-CN (G)	10	0.681	0.180	0.737	0.273	0.370	0.852
	F-NC	10	0.575	0.330	0.500	0.394	0.163	1.322
After	TNC-BF(G)	10	0.261	0.264	0.159	0.339	0.033	0.751
	TNC-BF	10	0.299	0.159	0.243	0.209	0.084	0.618
	TNC-CN (G)	10	0.279	0.341	0.145	0.414	0.021	1.004
	TNC-CN	10	0.097	0.099	0.074	0.138	0.017	0.328
	O-BF (G)	10	0.213	0.214	0.137	0.353	0.014	0.568
	O-BF	10	0.223	0.216	0.133	0.338	0.036	0.608
	O-CN (G)	10	0.262	0.408	0.119	0.256	0.016	1.377
	O-CN	10	0.134	0.161	0.069	0.162	0.015	0.506
	F-BF (G)	10	0.422	0.231	0.352	0.327	0.180	0.875
	F-BF	10	0.580	0.398	0.497	0.746	0.119	1.304
	F-CN (G)	10	0.261	0.163	0.195	0.244	0.015	0.540
	F-CN	10	0.286	0.263	0.199	0.289	0.046	0.907

*n* Sample, *SD* Standard deviation, *IQR* Interquartile range, *F-BF* Filtek Bulk Fill, *F-CN* Filtek Z250-XT, *TNC-BF* Tetric N-Ceram Bulk-fill, *TNC-CN* Tetric N-Ceram y, *O-BF* Opus Bulk Fill APS, *O-CN* Opallis EA2, *CN* Conventional Nanohybrid, (*G*) With oxygen inhibited layer control

Before polishing, when comparing surface roughness of all groups of bulk fill and conventional nanohybrid resin composites, with and without oxygen inhibition layer control, no significant differences could be observed (*p* = 0.308). However, after polishing, when comparing all groups of resin composites, significant differences could be observed in at least two of the groups (*p* = 0.002). Thus, when performing multiple comparisons of surface roughness, significant differences could be seen between Tetric N-Ceram resin composite and Filtek Bulk Fill resin with control (*p* = 0.023) and without control (*p* = 0.010) of the oxygen inhibited layer, being the latter significantly different from Opallis EA2 resin composite (*p* = 0.044). (Table 3).

**Table 3:** Comparison of surface roughness before and after polishing of bulk fill and conventional nanohybrid resin composites with and without oxygen inhibited layer control

Polish	Resin composite	*n*	Median	IQR	K–W	*p*-value
Before	TNC-BF(G)	10	0.547	0.692	12.776	0.308
	TNC-BF	10	0.503	0.740		
	TNC-CN (G)	10	0.466	0.477		
	TNC-CN	10	0.581	0.572		
	O-BF (G)	10	0.374	0.332		
	O-BF	10	0.669	0.830		
	O-CN (G)	10	0.514	0.483		
	O-CN	10	0.442	0.285		
	F-BF (G)	10	0.462	0.267		
	F-BF	10	0.660	0.711		
	F-CN (G)	10	0.737	0.273		
	F-CN	10	0.500	0.394		
After	TNC-BF(G)	10	0.159	0.339	29.007	0.002*
	TNC-BF	10	0.243	0.209		
	TNC-CN (G)	10	0.145	0.414		
	TNC-CN	10	0.074^a^	0.138		
	O-BF (G)	10	0.137	0.353		
	O-BF	10	0.133	0.338		
	O-CN (G)	10	0.119	0.256		
	O-CN	10	0.069^a,b^	0.162		
	F-BF (G)	10	0.352^b,c^	0.327		
	F-BF	10	0.497^c^	0.746		
	F-CN (G)	10	0.195	0.244		
	F-CN	10	0.199	0.289		

Different letters were used to indicate significant differences (*p* < 0.05) between independent pairs, according to Bonferroni post hoc adjustment. However, if two values coincide with equal letters it means that there were no differences between them

*n* Sample; *IQR* Interquartile range; *K–W* Kruskall–Wallis test, *p < 0.05: Significant differences in at least two groups. *F-BF* Filtek Bulk Fill, *F-CN* Filtek Z250-XT, *TNC-BF* Tetric N-Ceram Bulk-fill, *TNC-CN* Tetric N-Ceram y, *O-BF* Opus bulk fill APS, *O-CN* Opallis EA2; *CN* Conventional nanohybrid, (*G*) With oxygen inhibited layer control

**Fig. 8:**
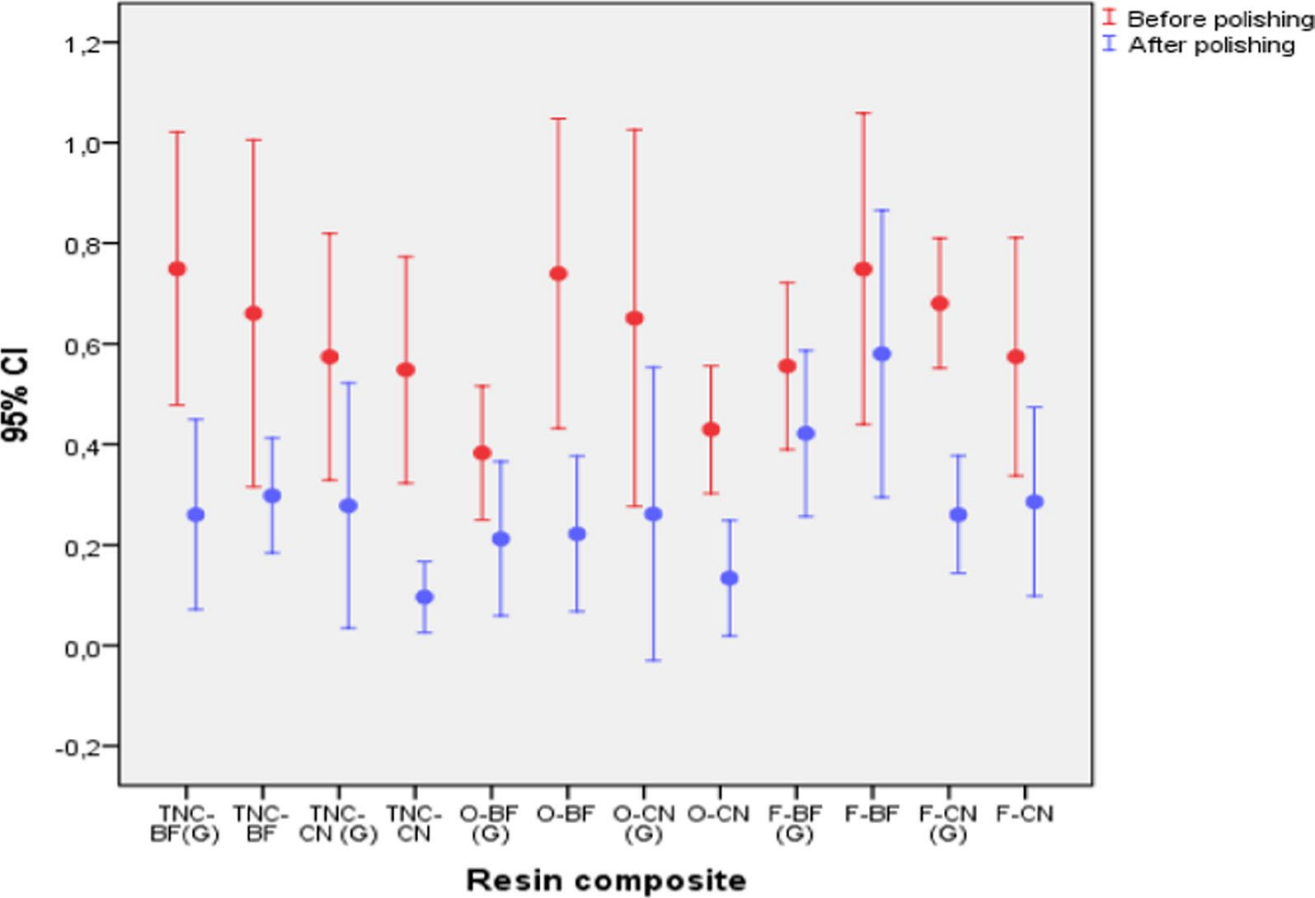
Comparison of average difference of surface roughness values between resin composite groups before and after polishing

When comparing the surface roughness variation between before and after (${\overline{\text{X}}}_{{\text{f}}} - {\overline{\text{X}}}_{{\text{i}}}$) polishing of bulk fill and conventional nanohybrid resin composites, with and without oxygen inhibited layer control, it could be observed that the surface roughness in all resin composite groups decreased significantly (*p* < 0.05), except for the Opus Bulk Fill APS resin composite with oxygen inhibited layer control (*p* = 0.125) (Fig. 8). On the other hand, when making comparisons of the variations between all groups of resin composites, significant differences could be observed in at least two groups (*p* = 0.021). However, when a post-test was performed with the Bonferroni adjustment, it was found that these differences between at least two groups were not significant for any comparison (*p* > 0.05). (Table 4).

**Table 4:** Surface roughness variation between before and after polishing of bulk fill and conventional nanohybrid resin composites, with and without oxygen inhibited layer control

Resin composite	(${\overline{\text{X}}}_{{\text{f}}} - {\overline{\text{X}}}_{{\text{i}}}$)	Median	SD	SE	95% CI		w	t	p*	K–W	p**
					LL	UL					
TNC-BF(G)	− 0.489	− 0.419^a^	0.243	0.077	− 0.663	− 0.315	0.060	− 6.353	0.000	22.462	0.021**
TNC-BF	− 0.362	− 0.254^a^	0.465	0.147	− 0.695	− 0.030	0.196	− 2.463	0.036		
TNC-CN (G)	− 0.296	− 0.272^a^	0.145	0.046	− 0.400	− 0.192	0.334	− 6.436	0.000		
TNC-CN	− 0.452	− 0.552^a^	0.266	0.084	− 0.642	− 0.262	0.227	− 5.384	0.000		
O-BF (G)	− 0.170	− 0.222^a^	0.319	0.101	− 0.398	0.058	0.917	− 1.691	0.125		
O-BF	− 0.518	− 0.504^a^	0.441	0.140	− 0.833	− 0.202	0.338	− 3.709	0.005		
O-CN (G)	− 0.389	− 0.412^a^	0.248	0.079	− 0.567	− 0.212	0.493	− 4.959	0.001		
O-CN	− 0.295	− 0.303^a^	0.201	0.064	− 0.439	− 0.151	0.518	− 4.640	0.001		
F-BF (G)	− 0.134	− 0.103^a^	0.110	0.035	− 0.213	− 0.056	0.192	− 3.877	0.004		
F-BF	− 0.169	− 0.188^a^	0.086	0.027	− 0.231	− 0.107	0.387	− 6.187	0.000		
F-CN (G)	− 0.420	− 0.430^a^	0.244	0.077	− 0.594	− 0.246	0.333	− 5.448	0.000		
F-CN	− 0.288	− 0.294^a^	0.212	0.067	− 0.440	− 0.136	0.886	− 4.294	0.002		

Equal letters (a) were used to indicate no significant difference (*p* > 0.05) for independent pairwise comparison, according to Bonferroni post hoc adjustment

(${\overline{\text{X}}}_{{\text{f}}} - {\overline{\text{X}}}_{{\text{i}}}$): Mean difference; (${\overline{\text{X}}}_{{\text{f}}}$): After polishing; (${\overline{\text{X}}}_{{\text{i}}}$): Before polishing; *SD* Standard deviation; *SE* Standard error of mean; *95% CI* 95% confidence interval, *LL* Lower limit, *UL* Upper limit; *w*: Normality analysis based on Shapiro Wilk Test (normal distribution: *p* > 0.05); *t*: Student’s t-test for related measures (significant differences *p < 0.05). *K–W* Kruskall Wallis test (significant differences in at least two groups: **p < 0.05)

## Discussion

Surface quality of resin composites is important because poor polishing could be detrimental by compromising their durability. In addition, control of inhibited oxygen layer is crucial as it could compromise the mechanical properties of resin composites [11, 17]. Therefore, the present study aimed to assess surface roughness of bulk-fill and conventional nanohybrid resin composites, with and without polishing, after controlling the oxygen inhibited layer. As a result, it was obtained that Bulk Fill resins (Filtek, Tetric N-Ceram and Opus APS) and conventional nanohybrid composite resins (Filtek Z250 XT, Tetric N-Ceram and Opallis EA2) after being polished with prior control of the oxygen inhibited layer, showed consistent and significant decrease in surface roughness, thus rejecting the null hypothesis.

Glycerin has been used in dentistry to control the oxygen inhibited layer (OIL). Oxygen inhibits polymerization because its reactivity with free radicals is greater than that of resin composite monomers. During this inhibition process, oxygen interacts with the resin liquid and is consumed by the formed radicals [2, 10, 16]. In this sense, glycerin converts the highly reactive radicals on the surface into relatively stable hydroperoxides, which allows to obtain a better light-curing quality in the outermost layer of resin composites, avoiding the formation of OIL [10, 17]. For this reason, in the present study it was decided to use glycerin because it avoids the contact of atmospheric oxygen with the surface of the resin composite, thus preventing it from reacting with free radicals and improving the degree of conversion and surface mechanical properties [2, 10]. Although studies such as Lassilla et al. [24] and Strnad et al. [25] suggest that celluloid tape controls OIL since it blocks the contact of the material with oxygen, they also reported that it would not eliminate it completely since it can trap bubbles during placement. Therefore, this study opted for additional use of glycerin.

In spite of the above, the results of present study showed no significant differences in roughness when analyzing resin composites with and without control of the oxygen inhibited layer, being in agreement with the results obtained by Tsujimoto et al. [26] However, this was discrepant with that obtained by Borges et al. [2] and Meita et al. [16], perhaps because they used resin composites with different chemical composition than the sample of present study, being this a determinant factor in surface roughness. [2, 10, 17]. In addition, the polishing system used by Borges et al. [2] and Meita et al. [16] was different from the one used in present study.

The polishing system used in present study was the Sof-lex disc, which is an abrasive disc impregnated with aluminum oxide. Its use was justified because it was reported as the system that presents the lowest surface roughness with respect to other commonly used systems [27]. However, it should be taken into account that surface roughness can also be related to other factors, for example: number of steps, polishing time, particle size of the organic load in resin composites, among others [2, 10, 15, 16]. Regarding the number of steps, Jones et al. [28] reported that for a multipass system, 25 s of polishing should be performed for each disc used. However, in accordance with the manufacturer's recommendations, in present study it was decided to apply 20 s of polishing per disc [29]. On the other hand, Kılıç et al. [30] reported that particle size of the organic filler in resin composite influences its surface roughness, and further reported that bulk fill resin composites exhibited higher roughness because they contain large filler particles to increase translucency while achieving composite application in a single 4 mm layer, unlike the nanohybrid resin composites that contain smaller filler particles, which reduces the interparticle spacing, limiting the removal of both particles and organic matrix during polishing and indirectly preventing an increase in surface roughness [30]. In this sense, in present study the Filtek Bulk Fill resin composite with and without OIL control presented higher surface roughness compared to the conventional Tetric N-Ceram nanohybrid resin composite after polishing. This could be related to particle size and filler components, as Tetric N-Ceram resin composite (0.5–1.5 µm) [31] has barium glass filled with ytterbium fluoride, while Filtek Bulk Fill resin composite (0.5–4 µm) [32] contains zirconium and silica within its composition [33]. However, the Opus Bulk Fill APS resin composite showed lower surface roughness than Tetric N-Ceram Bulk Fill and Filtek Bulk Fill before polishing, maintaining similar values after polishing with and without control of the oxygen inhibited layer. This was probably due to the fact that this resin composite works with a new APS (*Advanced Polymerization System*) technology patented by FGM, which consists of a combination of different photoinitiators that interact with each other and allow to amplify the polymerization capacity, increasing the degree of conversion and depth of cure, which allows us to suppose that this would improve the mechanical and surface properties [33, 34]. Additionally, it should be noted that a single polishing system will not produce the same effects on every type of resin composite, regardless of OIL control [12]. It is worth mentioning that Opus Bulk Fill APS resin composite with OIL control maintained its low surface roughness values before and after polishing, being different from when OIL was not controlled, since the values were significantly reduced after polishing. This may have occurred because the glycerin applied to the last layer of Opus Bulk Fill APS resin composite prior to light curing behaved as an atmospheric oxygen inhibitor, helping to convert the highly reactive radicals on the surface into relatively stable hydroperoxides, allowing for better light curing quality in the outermost layer [35].

In present study, the surface roughness of the conventional nanohybrid and bulk fill resin composites with and without OIL control did not exceed an average of 0.75 µm and 0.58 µm before and after polishing, respectively. These values are in agreement with the ISO 1302:2002 surface quality standard, [36] which considers surface roughness between 0.0025 and 0.8 µm as acceptable. Furthermore, the values obtained in present study agree with those obtained by Midobuche et al. [37] who assessed surface roughness of the Sof-Lex^®^ polishing system on esthetic nanoparticle resin composites, obtaining surface roughness values below 1 µm, which is acceptable within clinical parameters.

The present study is important because, considering the results obtained, surface roughness could be improved with a finishing and polishing procedure regardless OIL control or not. This allows to recommend finishing and polishing not only for aesthetic reasons, but also to improve the surface of both conventional nanohybrid and bulk fill resin composites, since it will significantly reduce the formation of grooves and irregularities on surface, with excellent polish and high gloss, avoiding the accumulation of plaque and pigmentations that could alter the natural appearance of the restoration, in addition to facilitating longevity of resin composite both aesthetically and in its functional performance [10, 38]. However, clinically, it is not easy to access all resin surfaces when polishing, so it is also suggested to apply glycerin before light curing the last layer to ensure good polymer conversion, avoiding the formation of the oxygen inhibited layer.

As a limitation of the present study, it is recognized that results obtained cannot be fully extrapolated to clinical practice since it is an in vitro study. In addition, it is important to highlight that the use of stainless steel metallic matrix to make the samples, as indicated by ISO 4049–2019, [23] could underestimate the depth of polymerization that actually occurs in a clinical situation, because the internal walls of the metallic matrix do not scatter the light but absorb it, reducing the amount of photons available for activation. [39, 40]

It is recommended for future studies to control the polishing time variable and check if it is an influential factor in the resin composite surface roughness. In addition, the oxygen inhibited layer and roughness could be evaluated by comparing different polishing systems and using resin composites with different composition, since this could be a determining factor in surface roughness.

## Conclusion

In summary, recognizing limitations of the present in vitro study, the Opus Bulk Fill APS resin composite with oxygen inhibited layer control presented lower surface roughness, both before and after polishing, being these values similar at both times. However, after polishing of the other bulk fill and conventional nanohybrid resin composites, with and without oxygen inhibited layer control, the surface roughness decreased significantly in all groups, being this decrease similar in all of them.

## Supplementary Information

**Additional file 1: Table S1.** Surface roughness data of resin composites with and without polishing, according to the oxygen inhibited layer control.

## Data Availability

The data recorded in this study are available as supplementary material in this paper.

## Abbreviations

Bis-GMA: Bisphenol-A-glycidyl methacrylate
Bis-EMA: Bisphenol A polyethylene glycol diether dimethacrylate
BAPO: Bisacyl phosphine oxide
BF: Bulk fill
CI: Confidence interval
CRIS: Checklist for reporting in-vitro studies
HEMA: Hydroxyethylmethacrylate
CN: Conventional nanohybrid
OIL: Oxygen inhibited layer
TEGDMA: Triethylene glycol dimethacrylate
UDMA: Urethane dimethacrylate
SPSS: Statistical package for the social sciences
