# Supplementary material for: Surface roughness and oxygen inhibited layer control in bulk-fill and conventional nanohybrid resin composites with and without polishing: in vitro study
Source: BMC Oral Health. 2022 Jun 26;22:258. doi: 10.1186/s12903-022-02297-w (PMC9235274; doi:10.1186/s12903-022-02297-w)
Supplement: Supplementary file 1 — Additional file 1: Table S1. Surface roughness data of resin composites with and without polishing, according to the oxygen inhibited layer control. [file 12903_2022_2297_MOESM1_ESM.pdf]

Supplementary table 1. Surface roughness data of resin composites with and without polishing, according to the oxygen inhibited layer control.

ID	Resin	Glycerin	surface roughness (µm) Before polishing	After polishing
1	1	1	1.221	0.751
2	1	1	0.485	0.159
3	1	1	0.592	0.160
4	1	1	0.501	0.094
5	1	1	1.148	0.159
6	1	1	1.441	0.727
7	1	1	0.492	0.327
8	1	1	0.441	0.127
9	1	1	0.774	0.070
10	1	1	0.403	0.033
11	1	2	0.970	0.360
12	1	2	0.395	0.084
13	1	2	0.576	0.386
14	1	2	0.120	0.248
15	1	2	0.489	0.158
16	1	2	1.498	0.217
17	1	2	1.436	0.467
18	1	2	0.202	0.238
19	1	2	0.516	0.618
20	1	2	0.408	0.210
21	2	1	0.544	0.021
22	2	1	1.288	0.765
23	2	1	0.264	0.027
24	2	1	0.311	0.026
25	2	1	0.484	0.254
26	2	1	0.693	0.333
27	2	1	0.390	0.101
28	2	1	1.042	1.004
29	2	1	0.448	0.189
30	2	1	0.279	0.066
31	2	2	0.803	0.177
32	2	2	0.623	0.019
33	2	2	0.112	0.017
34	2	2	0.734	0.150
35	2	2	0.538	0.017
36	2	2	0.828	0.023
37	2	2	0.363	0.088
38	2	2	0.260	0.061
39	2	2	0.170	0.086
40	2	2	1.056	0.328

Resin composite	
TNC-BF	1
TNC-CN	2
O-BF	3
O-CN	4
F-BF	5
F-CN	6

F-BF: Filtek Bulk Fill,
F-CN Filtek Z250-XT, Conventional Nanohybrid
TNC-BF: Tetric N-Ceram Bulk-fill,
TNC-CN: Tetric N-Ceram, Conventional Nanohybrid
O-BF: Opus Bulk Fill APS,
O-CN: Opallis EA2, Conventional Nanohybrid

Glycerin	
Yes	1
No	2

41	3	1	0.500	0.298
42	3	1	0.629	0.014
43	3	1	0.561	0.027
44	3	1	0.124	0.568
45	3	1	0.271	0.028
46	3	1	0.426	0.548
47	3	1	0.113	0.079
48	3	1	0.302	0.325
49	3	1	0.582	0.195
50	3	1	0.322	0.044
51	3	2	0.849	0.074
52	3	2	0.503	0.187
53	3	2	1.340	0.168
54	3	2	0.398	0.348
55	3	2	0.380	0.036
56	3	2	0.761	0.098
57	3	2	0.085	0.053
58	3	2	1.317	0.580
59	3	2	0.576	0.608
60	3	2	1.192	0.073
61	4	1	0.541	0.049
62	4	1	0.381	0.289
63	4	1	0.155	0.016
64	4	1	0.372	0.023
65	4	1	0.245	0.130
66	4	1	0.500	0.252
67	4	1	0.700	0.107
68	4	1	1.899	1.377
69	4	1	1.194	0.325
70	4	1	0.527	0.052
71	4	2	0.725	0.140
72	4	2	0.287	0.031
73	4	2	0.144	0.071
74	4	2	0.445	0.062
75	4	2	0.621	0.015
76	4	2	0.438	0.336
77	4	2	0.221	0.016
78	4	2	0.468	0.100
79	4	2	0.533	0.506
80	4	2	0.415	0.066
81	5	1	0.364	0.180
82	5	1	0.397	0.336
83	5	1	0.530	0.529
84	5	1	1.038	0.743
85	5	1	0.422	0.367
86	5	1	0.586	0.270
87	5	1	0.910	0.875

88	5	1	0.401	0.211
89	5	1	0.501	0.432
90	5	1	0.411	0.274
91	5	2	1.555	1.304
92	5	2	0.929	0.896
93	5	2	0.939	0.717
94	5	2	0.687	0.511
95	5	2	0.633	0.433
96	5	2	0.522	0.483
97	5	2	0.294	0.195
98	5	2	1.306	1.013
99	5	2	0.327	0.131
100	5	2	0.299	0.119
101	6	1	0.666	0.540
102	6	1	0.824	0.369
103	6	1	0.370	0.184
104	6	1	0.604	0.198
105	6	1	0.852	0.368
106	6	1	0.808	0.144
107	6	1	0.402	0.192
108	6	1	0.820	0.146
109	6	1	0.835	0.015
110	6	1	0.627	0.452
111	6	2	1.322	0.907
112	6	2	0.163	0.174
113	6	2	0.720	0.048
114	6	2	0.538	0.273
115	6	2	0.861	0.537
116	6	2	0.331	0.219
117	6	2	0.591	0.130
118	6	2	0.386	0.352
119	6	2	0.461	0.046
120	6	2	0.372	0.178
